# Invited comment on van der Steeg et al.: European consensus meeting of ARM-Net members concerning diagnosis and early management of newborns with anorectal malformations

**DOI:** 10.1007/s10151-015-1285-6

**Published:** 2015-02-28

**Authors:** David C. van der Zee

**Affiliations:** Department of Pediatric Surgery, Utrecht University Medical Center, Utrecht, The Netherlands

A conglomerate of European pediatric surgeons [1], united in the anorectal malformations network (ARMs-Net), refined the ‘Krickenbeck classification’ [2] that has served as a gold standard for the clinical management of anorectal malformations (ARM) over the past decade.

With expanding experience when dealing with ARM and continuing improvement in diagnostic and therapeutic means over the years, this is a welcome addition that deserves general support.

Regarding the differentiation between “experienced” surgeons and “not,” it is clear that ARMs are an infrequently occurring congenital anomalies and that extensive experience is not easy to acquire, if not in an ARM center. When to do a primary repair or when to perform a colostomy is clarified. However, this is also a signal that maybe in the future, patients with these pathologies should not be managed by everyone, but should be referred to specific centers that have a large experience.

The addition of temporary dilatation of the fistula in female patients with a fistula > H5 is refreshing. Particularly these days, when possible negative effects of anesthesia and/or surgery in neonates is in consideration, delay until the infant is somewhat older, yet before the onset of psychological sequelae, is a positive development. Therefore, temporary dilatation should not only be limited to premature infants or those with severe associated congenital anomalies, but may be appropriate for all infants.

Some restraint is warranted with some individual, yet unpublished, experience with puncturing the skin over the anus to determine the distance to the rectum. This is a highly subjective means of determining a distance that can be determined more objectively and repetitively by means of X-ray and/or ultrasound.
